# Acceptability to patients, carers and clinicians of an mHealth platform for the management of Parkinson’s disease (PD_Manager): study protocol for a pilot randomised controlled trial

**DOI:** 10.1186/s13063-018-2767-4

**Published:** 2018-09-14

**Authors:** Angelo Antonini, Giovanni Gentile, Manuela Giglio, Andrea Marcante, Heather Gage, Morro M. L. Touray, Dimitrios I. Fotiadis, Dimitris Gatsios, Spyridon Konitsiotis, Lada Timotijevic, Bernadette Egan, Charo Hodgkins, Roberta Biundo, Clelia Pellicano, on behalf of the PD_Manager consortium, Harry Hatzakis, Joao Correia, Angelo Antonini, Andrea Marcante, Roberta Biundo, Giovanni Gentile, Giglio Manuela, Luca Weis, Margherita Chiarot, Viviana Zanin, Barbara Korousic Seljak, Bojan Cestnik, Darko Aleksovski, Dragana Miljkovic, Franc Novak, Marko Bohanec, Tina Anzic, Vid Podpecan, Anita Valmarska, Gregor Papa, Bojan Blazica, Biljana Mileva Boshkoska, Robert Vilzmann, Francesca Assogna, Gianfranco Spalletta, Clelia Pellicano, Gaia Romana Pellicano, Valentina Palma, Cristina Scudellari, Tiziana Soru, Mino Napoletano, Francesca Fanciulli, Matteo Raffaelli, Adrian Banks, Brian Elliot, Charo Hodgkins, Ellen Seiss, Heather Gage, Lada Timotijevic, Bernadette Egan, Patrice Rusconi, Matthew Peacock, Sarah Gillies, Elizabeth Puttock, Morro M. L. Touray, Dimitris Gatsios, Dimitris Gatsios, George Rigas, Dimitrios Fotiadis, Cecilia Vera, Javier Uceda, Samanta Villanueva Mascato, Javier Rojo de la Cal, José Javier Serrano Olmedo, Fernando Martinez, Maria Teresa Arrendondo, Imre Cikajlo, Karmen Peterlin-Potisk

**Affiliations:** 1 Department of Neuroscience, University of Padua, Padua, Italy; 2 IRCCS San Camillo Hospital, Venice, Italy; 3 Surrey Health Economics Centre, Faculty of Health and Medical Sciences, University of Surrey, Guildford, GU2 7XH UK; 4 Department of Materials Science, Unit of Medical Technology and Intelligent Information Systems, University of Ioannina, Ioannina, Greece; 5 Department of Neurology, Medical School, University of Ioannina, Ioannina, Greece; 6 Department of Psychology, University of Surrey, Guildford, England; 7 Fondazione Santa Lucia IRCCS, Via Ardeatina 306, 00179 Rome, Italy; 8 Department of Neuriscience, Mental Health and Sensory Organs, Sapienza University, Via di Grottarossa 1035, 00189 Rome, Italy

**Keywords:** Parkinson’s disease, mHealth, Acceptability, Utility, Cost consequence analysis

## Abstract

**Background:**

Parkinson’s disease is a degenerative neurological condition causing multiple motor and non-motor symptoms that have a serious adverse effect on quality of life. Management is problematic due to the variable and fluctuating nature of symptoms, often hourly and daily. The PD_Manager mHealth platform aims to provide a continuous feed of data on symptoms to improve clinical understanding of the status of any individual patient and inform care planning. The objectives of this trial are to (1) assess patient (and family carer) perspectives of PD_Manager regarding comfort, acceptability and ease of use; (2) assess clinician views about the utility of the data generated by PD_Manager for clinical decision making and the acceptability of the system in clinical practice.

**Methods/design:**

This trial is an unblinded, parallel, two-group, randomised controlled pilot study. A total of 200 persons with Parkinson’s disease (Hoehn and Yahr stage 3, experiencing motor fluctuations at least 2 h per day), with primary family carers, in three countries (110 Rome, 50 Venice, Italy; 20 each in Ioannina, Greece and Surrey, England) will be recruited. Following informed consent, baseline information will be gathered, including the following: age, gender, education, attitudes to technology (patient and carer); time since Parkinson’s diagnosis, symptom status and comorbidities (patient only). Randomisation will assign participants (1:1 in each country), to PD_Manager vs control, stratifying by age (1 ≤ 70 : 1 > 70) and gender (60% M: 40% F). The PD_Manager system captures continuous data on motor symptoms, sleep, activity, speech quality and emotional state using wearable devices (wristband, insoles) and a smartphone (with apps) for storing and transmitting the information. Control group participants will be asked to keep a symptom diary covering the same elements as PD_Manager records. After a minimum of two weeks, each participant will attend a consultation with a specialist doctor for review of the data gathered (by either means), and changes to management will be initiated as indicated. Patients, carers and clinicians will be asked for feedback on the acceptability and utility of the data collection methods. The PD_Manager intervention, compared to a symptom diary, will be evaluated in a cost-consequences framework.

**Discussion:**

Information gathered will inform further development of the PD_Manager system and a larger effectiveness trial.

**Trial registration:**

ISRCTN Registry, ISRCTN17396879. Registered on 15 March 2017.

**Electronic supplementary material:**

The online version of this article (10.1186/s13063-018-2767-4) contains supplementary material, which is available to authorized users.

## Background

Parkinson’s disease is a degenerative neurological condition associated with a range of motor and non-motor symptoms which have a very serious effect on the quality of life of the people affected. The processes that lead to Parkinson’s, and to how it manifests in individuals, involve numerous variables and pathways, and the aetiology is still not fully understood. Hence, management of the condition is challenging; people with Parkinson’s differ significantly in their symptoms and severity, how their disease progresses, their responses to treatments and their risk of complications [1]. To optimise treatment, a personalised approach is therefore needed [2].

A particular characteristic of advanced Parkinson’s disease is the fluctuation of symptoms and on/off periods. The mainstay of management is a pharmacological regimen that becomes increasingly complex as the disease progresses. Clinicians often find it difficult to identify the appropriate combination of medications because the clinical examination is just a snapshot of the patient’s fluctuating state. To accurately titrate the doses, they therefore have to rely on reports by patients and carers during short and infrequent clinic appointments. Electronic and paper motor symptom diaries have been used to improve the information available to clinicians. Both forms of reporting have, however, been shown to have similar numbers of erroneous entries, and a need for detection using automatic wearable devices has been identified [3]. More continuous symptom evaluation and feedback to clinicians could provide the required accurate and reliable information, with the potential to improve treatment and outcomes. This is the rationale behind the development of the PD_Manager system. This mHealth platform aims to provide a continuous feed of data on symptoms to improve clinical understanding of the fluctuating status of any individual patient and to inform care planning in which different experts may be involved, as well as prescribing. Continuous quantitative monitoring of activities and medication-induced fluctuations using wearable devices has been found feasible and useful in prior studies [4, 5], including exercise interventions [6]. Research has also focussed on finding the optimal location for monitoring motor performance [7].

The whole PD_Manager project involves a consortium of partners from several European countries. The development of the PD_Manager platform has been informed by qualitative research (interviews and focus groups) which explored the views and attitudes of people with Parkinson’s, carers and health professionals on the use of the technology for symptom control. Researchers have worked with device developers to refine the technology and undertake proof of concept testing in a hospital setting in Italy and Greece. No adverse events were experienced, and the pilot trial reported in this paper represents the next step in the testing of the system. The processes of the PD_Manager system will be checked in a community setting, and its acceptability and utility in clinical practice will be explored. Unlike other ongoing tests of wearable sensors in Parkinson’s patients which are observational [8], the PD Manager pilot will use a randomised controlled design. The most common traditional way of gathering information on symptoms from patients is through patient diaries [9]. This pilot study will therefore use symptom diaries as a comparator to the automated and electronic PD_Manager system for informing patient care and treatment plans.

### Research questions

The research questions are the following:

1. What is the utility of PD_Manager as an aid to clinical decision making (in terms of informing patient care and treatment plans)?
2. How acceptable is PD_Manager to patients and carers?
3. What would be the resource implications of the use of the PD_Manager platform in healthcare programmes from a provider’s perspective?

### Aims and objectives

The aim of the pilot study is to explore the acceptability and utility of the PD_Manager system to patients, carers and clinicians, in a community setting, compared to a symptom diary, to inform further developments and the design of a larger clinical trial which will assess effectiveness (patient and carer health outcomes).

The specific objectives of the pilot study are to:

1. Assess patient perspectives of the PD_Manager mHealth platform regarding comfort, acceptability, ease of use and understanding of Parkinson’s
2. Assess the views of clinicians about the PD_Manager mHealth platform, i.e. usefulness of the information provided for decision making regarding patient management; acceptability in clinical practice; confidence in reliability of the information
3. Explore the resource implications and costs of the PD_Manager system for patients/carers and providers and consider these in the light of potential outcome differences, compared to the symptom diary, in a cost-consequences framework.

## Methods/design

### Design

An unblinded, parallel, two-group randomised controlled pilot study will be conducted to assess the acceptability and utility of the PD_Manager system, compared to traditional practices of using a symptom diary, for the management of people with Parkinson’s disease. The trial will be undertaken in three countries (England, Greece and Italy). The protocol and all study documents were prepared in English and subsequently translated into Greek and Italian.

### Participants

A total of 200 people with Parkinson’s and their carers (recruited as dyads) will be enrolled into the study through clinical centres (110 Rome, 50 Venice, Italy; 20 each in Surrey, England and Ioannina, Greece).

The inclusion criteria for people with Parkinson’s are as follows: (1) diagnosis of idiopathic Parkinson’s disease according to the UK Brain Bank Criteria; (2) Hoehn and Yahr disease stage 3 or 4 in OFF state [10]; (3) presence of motor fluctuations with an average of at least 2 h of OFF state during the day; (4) availability of a live-in carer who is willing to take part in the study; (5) good understanding of local language (in order to be able to complete the research). The exclusion criteria for people with Parkinson’s are the following: (1) presence of severe cognitive impairment (Parkinson’s disease dementia); (2) comorbidities with stroke or other brain disease; (3) current involvement in other Parkinson’s research.

For the assessment of the clinical perspective, a minimum of ten neurologists/prescribing clinicians will be asked to participate (six in Italy, two each in England and Greece).

### Trial processes

**Fig. 1:**
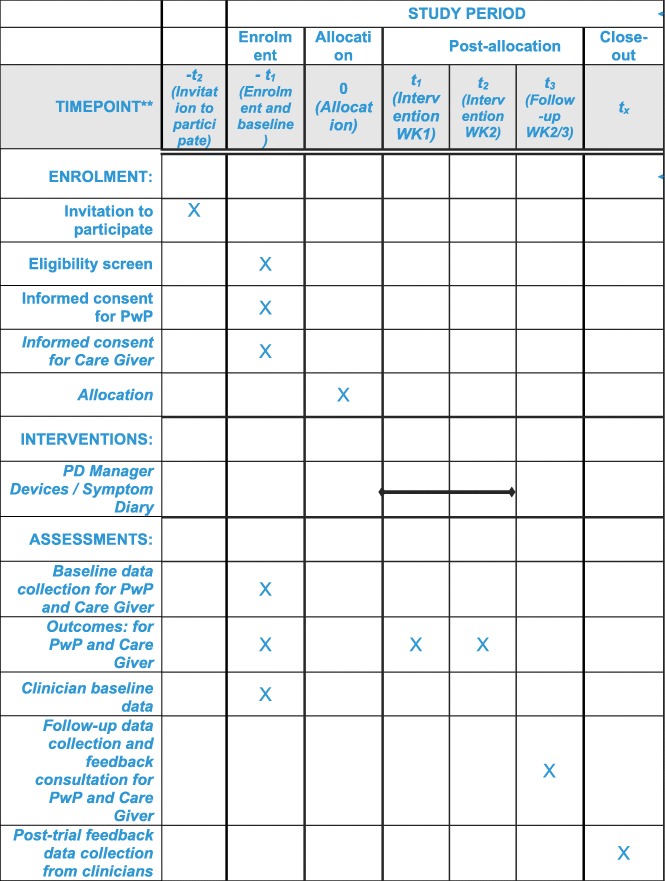
SPIRIT figure of enrolment, interventions and follow-up assessments

**Fig. 2:**
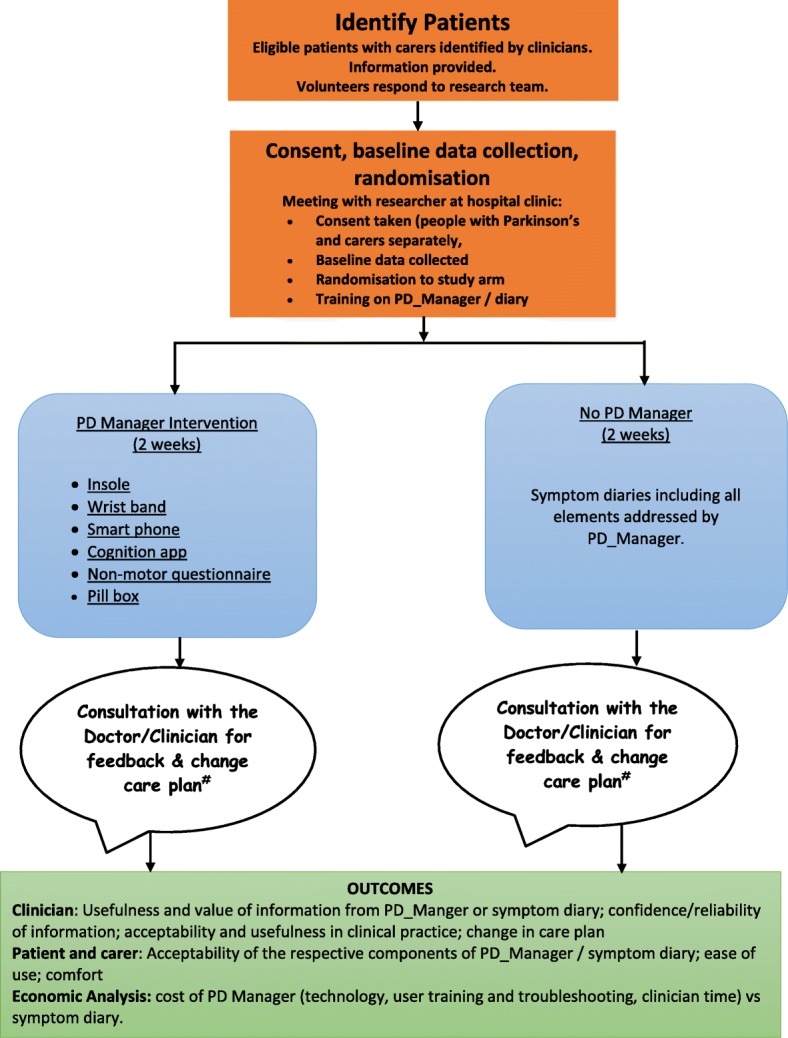
PD_Manager schematic diagram of trial process

The Standard Protocol Items: Recommendations for Interventional Trials (SPIRIT) checklist is provided as Additional file 1. Patient flow through the trial is shown schematically in Fig 1; the SPIRIT checklist is summarised in Fig 2. Data collection at each stage is shown in Table 1.

**Table 1:** Summary of data collection at each stage

Participant group		Data capture at each stage		
	Baseline	During intervention, PD_Manager group from devices	During intervention, control group symptom diaries	Post-intervention, 2 week follow-up
Patient	Age, gender, education, disease duration, disease stage (Hoehn and Yahr score), main symptoms (tremor, bradykinesia, rigidity, dyskinesia), more affected side, UPDRS scores, current medications, comorbidities, views on technology Outcomes: EQ-5D-5L; PDQ-8; NMSS	Motor symptoms (gait, freezing of gait, bradykinesia, hypokinesia, dyskinesia, exercise); non-motor symptoms (cognition, sleep, mood)	All symptom domains captured through the PD_Manager intervention were self reported in a motor diary [16] and a wellbeing map [17] including: on/off; speech; sleep challenges; cognitive issues; activities and physiotherapy	Interviews on acceptability and ease of use of PD_Manager or symptom diary; usefulness of the education section of PD_Manager Outcomes: EQ-5D-5L; PDQ-8 and NMSS Changes in management plan and referrals
Caregiver	Age, gender, education, views on technology	No information is collected from caregivers in the PD_Manager group	No information is collected from clinicians in the symptom diary group	Interviews on acceptability and ease of use of PD_Manager or symptom diary; usefulness of the education section of PD_Manager
	Outcome: Zarit Caregiver Burden Scale (using short version)			Outcome: Zarit Caregiver Burden Scale (using short version)
Clinician	Qualifications, current role, number of Parkinson’s patients/week, length of time in practice, views on technology	No information is collected from clinicians in the PD_Manager group	No information is collected from clinicians in the symptom diary group	Usefulness and value of the information gathered (PD_Manager and symptom diary) for influencing management decisions; changes in management, referrals made

*EQ-5D-5L* EuroQoL-5Dimenions-5Levels, generic health-related quality of life scale [18–20], *PDQ-8* Parkinson’s Disease Questionnaire-8, disease specific measure of health-related quality of life [21, 22], *NMSS* Non-Motor Symptoms Scale [23], *UPDRS* Unified Parkinson’s Disease Rating Scale [11]

#### Recruitment, consent and baseline data collection of patient/carer dyads

People with Parkinson’s and their live-in carers will be identified by the consultant neurologist at each centre during routine clinic appointments or following an inpatient episode and invited to take part in the study. Eligibility will be confirmed by the clinician through completion of an eligibility checklist (based on the inclusion and exclusion criteria). The consultant will explain the purpose of the study and provide an information sheet containing full details of the study (see Additional file 2). The dyads will be given time to consider and asked to contact the research team if they want further information or wish to participate. Those who volunteer will be given an appointment to attend the hospital to meet a research nurse, where consent will be taken (separately by the person with Parkinson’s and the carer — see Additional file 2) and baseline data collected (from records, interview and clinical examination). Participants will be given unique study identifiers to maintain anonymity.

Baseline data collected (see Table 1) for the person with Parkinson’s will include disease duration, disease rating — Unified Parkinson’s Disease Rating Scale (UPDRS) score [11], disease stage [10], side of onset, current symptoms (tremor, bradykinesia, rigidity, dyskinesia), more affected side, Parkinson’s medications, comorbidities and body mass index (BMI) calculated from ‘height and weight’. Age, gender, ethnicity, education level and comorbidities will be collected for both members of the dyad. In addition they will both be asked if they have a preference for either the PD_Manager system or the symptom diary and will be questioned regarding their attitudes and expectations for each approach (using the Technology Acceptance Model measure [12, 13] and the Global Attitude to Technology question [14]), as these attitudes may affect the views they will express after the trial.

#### Randomisation

Following baseline data collection, participants will be randomised to receive either PD_Manager (the intervention) or to be in the control group (symptom diary/no PD_Manager). Randomisation will be 1:1, blocked and by quota within each country and will be conducted using sealed envelopes prepared by an independent statistician. Given the higher incidence for Parkinson’s in men than in women, its increasing prevalence with age and a concern that younger people may be more accepting of technology than older people, the objective is to recruit 40% women and to have one half of each gender be under 70 years of age.

Participants will be informed of their group allocation immediately, and a researcher will provide the PD_Manager devices (intervention group) or the symptom diary (control group) and give full information about how they are to be used over the following 2-week trial period. Blinding of participants, researchers and clinicians will not be possible given the nature of the intervention.

#### Recruitment of clinicians

Volunteers will be sought from amongst clinicians recruiting patients to the study. They will be provided with an information sheet (see Additional file 2) and asked to provide informed consent (see Additional file 2). Baseline information will be gathered using a short questionnaire covering qualifications, current role, number of Parkinson’s patients treated per week and length of time in practice. Participants will also be asked to complete selected items from the Technology Acceptance Model measure [12, 13] and the Global Attitude to Technology question [14].

### PD_Manager mHealth intervention

PD_Manager devices comprise a wristband (Microsoft Band, Microsoft Corporation, Redmond, WA, USA), a pair of sensor insoles (Moticon GmbH, Munich, Germany), a smartphone (Aquaris M and U models, BQ, Madrid, Spain) and a knowledge platform (hosted by Biotronics 3D, London, UK). The devices are unobtrusive. Their wearability, sensitivity and reliability were tested as part of an earlier proof of concept study, as was the cleaning and sterilising process that occurs before devices are transferred between patients [15]. The intervention will be technically supported by the R&D partners involved in developing the devices, apps and knowledge management platform. Researchers will receive training in use of the devices.

Data that will be captured include:

- Motor symptoms (tremor, abnormal gait pattern, freezing of gait, body bradykinesia and dyskinesia), captured with the sensor insoles, wristband and phone accelerometers and gyroscopes, 24/7
- Activity data, including time spent on motor exercising, from the wristband, 24/7
- Speech quality (sound analysis, phonatory deficit) captured during scheduled task with the smartphone microphone (only at the Italian sites)
- Speech analysis (dysarthria, qualitative analysis of spontaneous speech, mood, intelligibility) through a web platform (only at the Italian sites)
- Depression, impulsivity and mood, through questionnaires on smart phone apps (as prompted by messages)
- Cognitive status through a battery of cognitive games on tailored apps.

The PD_Manager platform includes an education section containing videos and animations on symptoms and tips and other relevant information regarding personal care and how to cope with daily life challenges.

**Fig. 3:**
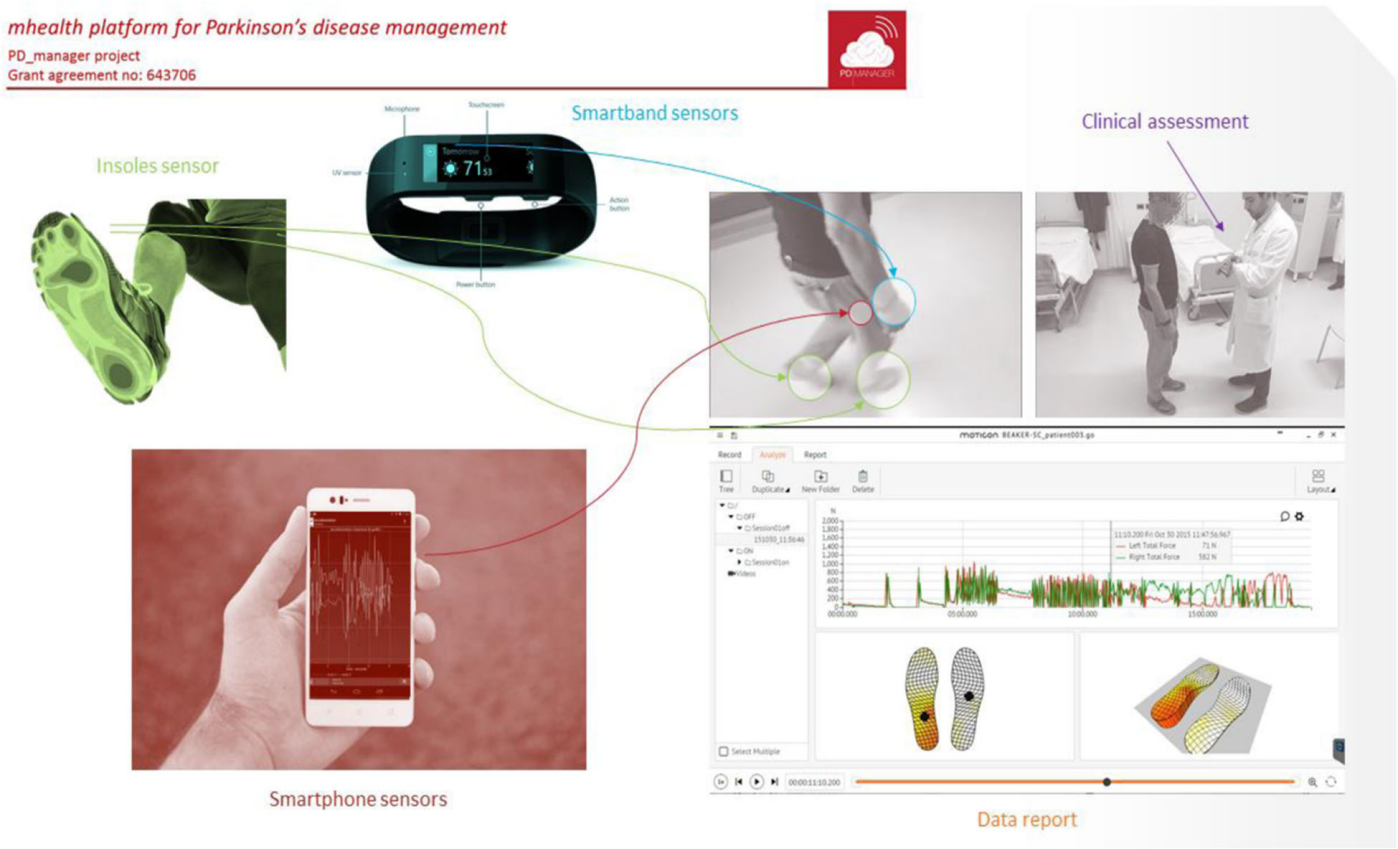
Devices used, body placement and preview of motor data collected

The intervention will last 14 days, which is considered sufficient time to monitor fluctuations for the purpose of planning treatments. Data from the PD_Manager devices and apps will be collected. The smartphone automatically transfers data to the cloud, including the data from the wristband (which is connected to the smartphone). Data is stored in the insoles and will be downloaded by the researcher in encrypted form at the end of the 2-week follow-up period and immediately transferred to the cloud. The devices used, body placement and a preview of motor data collected are shown in Fig. 3.

### Control group

Participants in the control group will be asked to record motor symptoms and wellbeing over the 2-week trial period. Motor symptoms will be captured using the Hauser diary, a validated instrument that allows the patient to record, in a dedicated form, his or her motor state every 30 min [16]. This diary is the current gold standard to monitor a patient’s symptoms at home, and it is also frequently used in pharmacological clinical trials to assess drug efficacy. The UCB Parkinson’s Well Being Map™ [17] allows patients to record and monitor the wide spectrum of Parkinson’s symptoms, including all those covered by PD_Manager. It is used to assist preparation for consultations with the healthcare team. Data from diaries will be manually entered into the trial database for analysis.

#### Concomitant care

All other usual care for participants will continue during the 2-week trial. The primary care doctors of all people with Parkinson’s will be informed in writing about their patient’s participation in the pilot study and the treatment arm to which they have been allocated.

### Follow-up

Participants in both groups will receive a phone call from the research fellow on day 5 and day 10 to check on progress and answer any questions. They will meet the clinician at the end of the 2-week trial period for a consultation guided by the data that have been collected. The follow-up appointment will be arranged during the baseline meeting, at a time convenient to participants, and will take place in a clinic at the hospital.

In the intervention group, the clinician reviewing each patient will receive reports with relevant information and management suggestions from the PD_Manager software. In the control group, the content of the patient symptom diaries will be reviewed. If data from either PD_Manager or the symptom diary indicate that management changes are required or referrals to other therapists (such as speech and language therapist, dietician, occupational therapist, physiotherapist) would be beneficial, this will be implemented.

### Acceptability outcomes

Assessment of acceptability for patients and carers will be undertaken at the end of the 14-day intervention period immediately after the feedback consultation with the clinician. A private room in the clinic will be identified for this purpose. Views will be collected by interview. Those in the intervention group will be asked about the following: how comfortable the devices were and how easy they were to use (determined with the same wearability and usability questions used in earlier testing of devices [15]; how useful the feedback they received from PD_Manager was; their views on communications with their neurologist regarding the PD_Manager outputs. The usefulness of the education section will also be assessed. A separate questionnaire will be used for participants in the control group to gather their views about the ease, burden and usefulness of the symptom diary. Questionnaires can be obtained from the corresponding author.

Back end data from the PD_Manager system will be provided to the researchers to enable an analysis of compliance with use of the devices by members of the intervention group. These data will be used to indicate how much the patients/carers valued using the devices.

Face-to-face interviews will be conducted with each clinician to explore their views about the relative usability and acceptability of PD_Manager and the symptom diary, and the value they perceive they provided in generating information for clinical decision making. Feedback will be sought by means of a semi-structured interview schedule on perceived usefulness to patients, carers and professionals, and adaptability to usual working schedules. The treating clinicians will be asked to comment on the ease of interpreting the data generated by the devices and the symptom diary and the ease of using this data in changing the treatment and care management of participants, as well as their views on this means of communicating with participants. The interview schedule can be obtained from the corresponding author.

### Feasibility assessment of effectiveness outcomes

As a test of feasibility, data collection at baseline and follow-up will include potential outcomes that might be used in a subsequent fully powered effectiveness trial. Outcome measures for people with Parkinson’s to be used are:

- EQ-5D-5L (EuroQoL-5Dimensions-5Levels), a commonly used generic measure of health-related quality of life across five domains (mobility, usual activities, self-care, pain, anxiety/depression), each scored on a 5-point scale (no problem to severe problem/unable) that provides a utility index for use in economic evaluations [18–20]
- PDQ-8 (Parkinson’s Disease Questionnaire-8), the short form of a Parkinson’s disease-specific measure of health-related quality of life covering eight domains scored on a 5-point scale (never to always/cannot do at all) [21, 22]
- NMSS (Non-Motor Symptoms Scale) for Parkinson’s, covering severity and frequency of 30 items across nine domains [23]

Live-in carers will be asked to complete the short version of the Zarit Caregiver Burden Scale consisting of 12 items, in two domains: personal strain and role strain. Each question is scored on a 5-point Likert scale (0, never to 4, almost always), giving a range of summed scores 0–48, with higher score representing a higher feeling of being burdened [24].

### Analysis

Reporting of results will follow Consolidated Standards of Reporting Trials (CONSORT) extension guidelines for randomised pilot and feasibility studies [25]. Data relating to individual participants will be recorded using unique study identifiers to maintain confidentiality. Research data will be analysed in each country and merged for comparison purposes. Data relating to quantitative variables (baseline characteristics of participants, acceptability and usability outcomes at follow-up) will be entered into Statistical Package for Social Sciences (SPSS) version 24 (IBM SPSS Statistics for Windows, version 24.0, IBM Corporation, Armonk, NY, USA) and analysed with simple frequencies; the intervention and control groups will be compared using appropriate statistical approaches, depending on the nature of the data. In accordance with the pilot nature of the study, the analysis will be non-confirmatory. Data from quality of life and other health outcomes will be inspected for potential use in any future effectiveness trials. Responses to open questions relating to participants’ views will be subject to narrative review. Two independent researchers will read and re-read comments and agree on themes within the data. The information generated by the PD_Manager devices will be analysed by the partners who developed each device.

We expect most participants to complete the trial, since the follow-up lasts only 2 weeks and ends with a consultation with a neurologist, that is in addition to their usual care. However, it is possible that a participant will be unable to complete or may wish to withdraw from the study, in which case their baseline data will be ignored. Another issue is that participants in the intervention group may not use the technology. We will know this from the automatic collection of data, and we will investigate reasons in the follow-up interview. Similarly, participants in the control group may not complete the diary, in which case we will similarly explore their reasons.

### Sample size calculation

A formal sample size calculation is not normally required for a pilot study [26–29]. Authors have suggested various figures ranging from 20 to 70 participants [26, 29–32]. The *n* = 20 value for number of subjects who will be included in England and Greece is at the lower end of this range, but there will be a total of 200 people with Parkinson’s across the three countries, 100 of whom will test the PD_Manager system. The sample size of 200 for the three countries was selected based on complexities around the technologies involved, the desire to compare men and women and different ages and the desire to capture possible cross-country setting variations. The findings from the pilot study will be used to inform the calculation of a sample size for a future larger scale trial.

### Economic evaluation

An economic analysis embedded in the trial will compare the PD_Manager intervention with the symptom diary with respect to costs and acceptability and usability outcomes in a cost-consequences framework [33]. The cost of providing, maintaining and insuring the PD_Manager devices, access to the cloud programme and the data infrastructure will be obtained from the manufacturers and averaged over the expected number of users. The amount of provider time spent in training patients and carers, troubleshooting and in interpreting feedback for both groups will be gathered through observation and interview and valued using nationally validated unit costs [34]. The time patients and carers report spending in using either the PD_Manager devices or the symptom diaries will be gathered from the questionnaires administered at follow-up. The various elements of costs and consequences for patients, carers and clinicians will be presented in a disaggregated form in a descriptive table. Reporting will follow Consolidated Health Economic Evaluation Reporting Standards (CHEERS) [35].

## Discussion

When testing of the PD_Manager system is complete and a final version becomes available for routine use in clinical practice, it is expected to be used to assess people with Parkinson’s with symptoms that are hard to manage and in circumstances where a clinician wants more information than is provided by patient/carer self-report to guide medication and management planning. This pilot study represents a relatively early stage in the PD_Manager development process, to test acceptability and utility of the system from patient, carer and clinician perspectives. The people with Parkinson’s who are recruited to the trial may not have symptoms that fit the description of ‘hard to manage’ and may, in the opinion of the recruiting doctors, already be optimally managed. However, their opinions about the acceptability and potential usefulness of the PD_Manager system will still be valuable. All participants will be reimbursed for all reasonable travel and other out-of-pocket costs that they incur in order to take part in the research.

The products that constitute the PD_Manager system in this trial have previously been tested by their respective manufacturers and shown to be in conformity with relevant directives of the European Commission including health, safety and environmental protection (i.e. they hold CE (Conformité Européene/European Conformity) certificates). The PD_Manager project also tested the devices for wearability, possible discomfort and safety as part of earlier work packages [15]. The participants in these earlier studies were carefully selected to represent the disease characteristics of the target group. Given the cost and complexity of the system, devices are reused for multiple patients, and cleaning instructions provided by the manufacturer are scrupulously followed to prevent cross infection. None of the devices were found to pose any risks or significant discomfort to the participants [15].

The pilot study has been designed to avoid standard risks of bias wherever possible. Participants will be randomised to the intervention and control groups to protect against selection bias, and care has been taken to ensure both groups receive the same attention at all stages of the study. Attrition or non-compliance (with devices or symptom diaries) do not constitute an issue but rather are actions of interest in our analysis of acceptability. However, the nature of the intervention is such that clinicians cannot be blinded to the group allocations. Potential does exist for selective outcome reporting. Hence, we are gathering information on attitudes to technology at baseline from participants, carers and clinicians.

The pilot study will not evaluate effectiveness and cost effectiveness of the PD_Manager system, although such a trial may be warranted if feedback from this pilot is positive. Outcome measures that may be used in an effectiveness trial are being tested within the pilot, and the findings will provide a basis for sample size calculation for a definitive trial. Preliminary cost estimates will also be computed in the pilot study to provide initial indications about the value of gathering further evidence from a larger trial.

Data security and confidentiality are major issues in trials of this sort. Data Protection Directive 95/46/EC, on the protection of individuals with regard to the processing of personal data and on the free movement of such data, will be strictly followed. Data from devices will be transferred (as described earlier) and stored in a web-based cloud database (NoSQL Database -3Dnet) in anonymised and encrypted format as employed by Apple for iPhones (a FiWare cloud-based process). The servers storing the information in the cloud platform are based on Biotronics 3D’s 3DnetMedical platform in the UK in an ISO27001-accredited data centre located in London. They are operated in accordance with the Data Protection Act. No third parties can access data either from the devices or during transmission. Access to the database will be strictly controlled and will only be shared with researchers on the PD_Manager project. Administrators of the web-based cloud database will not have access to data. The full data management policy of the cloud administrators is available from the corresponding author.

Data collected from participants by researchers will be recorded by unique study identification number. No names or personal information will be stored on or with these forms. Information will be kept in locked filing cabinets and password-protected computers in restricted access rooms at the study sites. Data will be shared only with researchers in the PD_Manager project. All data gathered will be the property of the PD_Manager consortium, and access by external researchers will only be authorised by the PD_Manager board. An embargo will be present until the end of the project with respect to access for researchers external to the study group.

Results of this pilot study will be presented to the funders as a report. The research team will write papers for publication in journals and make conference presentations to influence the development of future treatment and services for people with Parkinson’s. A summary of the study results will be available from the project website (http://www.parkinson-manager.eu/).

## Trial status

The protocol version number and date are version 4, 4 May 2017. Recruitment began on 17 October 2017 and will end on 31 March 2018. Note that recruitment had not completed when the paper was submitted.

## Additional files

Additional file 1: SPIRIT 2013 checklist: recommended items to address in a clinical trial protocol and related documents*. (DOC 121 kb)

Additional file 2: Patient, carer and clinician information sheets and consent forms. (DOCX 316 kb)

## Abbreviations

EQ-5D: EuroQOL-5 Dimensions, a standardised instrument for use as a measure of health outcome
MMSE: Mini Mental State Examination
NHS: National Health Service, UK
PD: Parkinson’s disease
PDQ: Parkinson’s Disease Questionnaire
SPSS: Statistical Package for Social Sciences
UPDRS: Unified Parkinson's Disease Rating Scale
