# Supplementary material for: Acceptability to patients, carers and clinicians of an mHealth platform for the management of Parkinson’s disease (PD_Manager): study protocol for a pilot randomised controlled trial
Source: Trials. 2018 Sep 14;19:492. doi: 10.1186/s13063-018-2767-4 (PMC6138904; doi:10.1186/s13063-018-2767-4)
Supplement: Supplementary file 2 — Patient, carer and clinician information sheets and consent forms. (DOCX 316 kb) [file 13063_2018_2767_MOESM2_ESM.docx]

Appendix B(i)

**PD_Manager. Pilot testing of devices to assist with symptom management for people with Parkinson’s.**

**Information sheet for people with Parkinson’s and family members or friends that live with them.**

We are inviting people with Parkinson’s and the family members or friends that live with them to take part in a research study. Before you decide, please take the time to carefully read the information below. This explains why the research is being done and what taking part would involve for you.

If you decide you would like to volunteer to take part, or would like further information about the study, details of how to contact the research team are given at the end of this information sheet.

**What is the PD_Manager project?**

People with Parkinson’s have different and sometimes severe symptoms. This affects their quality of life and makes it difficult to decide on the best treatment.

New technologies have been designed in the PD_Manager project to track Parkinson’s symptoms in real time and provide a continuous record for health professionals. We want to find out if this information can help doctors and Parkinson’s nurses to manage peoples’ symptoms better. The technologies include wristbands and insoles. The devices are linked to a smartphone which will be loaded with some ‘apps’ designed to help you manage your condition. The smartphone will also be used to store information about your symptoms and to send it to your doctor or Parkinson’s nurse.

PD_Manager is funded by the European Commission through the Horizon 2020 Research and Innovation programme. Researchers from six European countries are involved.

**What is the purpose of this study?**

The purpose of this pilot study is to test the PD_Manager system with a small number of people with Parkinson’s. We want to see if people with Parkinson’s and the family member or friend they live with find the devices acceptable. We will also ask doctors if they find the information that is generated through PD_Manager helpful when they are deciding on treatments. To help us assess how useful the PD_Manager system is, we are comparing it with a symptom diary.

**Why have we been invited to take part?**

You have been invited to participate in the pilot study because you are a person living with Parkinson’s and you experience the symptoms that the PD_Manager system monitors. Also someone lives with you who may help with the devices and who can also tell us how useful they think the system is.

People with Parkinson’s will not be able to take part unless a family member or friend that lives with them is also willing to be involved.

**Do we have to take part?**

Participation in this study is completely voluntary. The usual care and treatment of the person with Parkinson’s will not be affected in any way if you choose not to participate. You are able to withdraw at any point with no effect to the care you receive.

**What will happen if we take part?**

When you volunteer to participate in the pilot study, you will be asked to attend your hospital clinic where a Parkinson’s specialist nurse will ask you both (the person with Parkinson’s and the family member or friend that lives with you) to sign a form indicating your agreement to participate. The nurse will then collect some background information from you and your family member / friend, and also ask about your Parkinson’s symptoms. You will then be assigned to receive either the PD_Manager devices or the symptom diary. You will not be able to choose which group you are in. This will be determined by chance.

People in the PD_Manager group will be asked to wear a wristband and an insole in their shoes, and to use a smartphone on a daily basis for two weeks. You will be given the devices (including a PD Manager Smartphone) at the clinic and shown how to use them by a research technician. We will provide some written information for you to take home with you, and a telephone number that you can call if you have any questions during the two week trial period. The devices are designed to automatically collect data on the symptoms you experience and this will be made available to health professionals to help them decide on your care and treatment. The smartphone will contain some apps to provide relevant information to you.

If you are not allocated to the PD_Manager group, you will be given a specially designed diary. In this diary you will be asked to record the symptoms you experience on a daily basis for two weeks. All necessary instructions on how to complete the diary will be provided.

At the end of the two week data collection period, we will ask you to return to the clinic for a consultation with your doctor. The day and time of the consultation will be agreed with you during the initial visit with the nurse. You will be asked to bring the PD_Manager devices or the symptom diary with you to the consultation. The doctor will review the information in the devices or symptom diary and decide, in consultation with you, if any changes in your treatment might be needed. A researcher will ask you both (person with Parkinson’s and family member or friend) for your opinions about the devices or the diary. You will not be able to keep the devices or the diary after the consultation.

All reasonable travel expenses to both the original clinic visit and the follow up consultation will be refunded to you.

**Will our General Practitioner (GP) be involved?**

We will seek the permission of the person with Parkinson’s to inform their GP that they are taking part in this study, and the group you are allocated to.

**What are the possible risks of taking part?**

We are not aware of any risks associated with the devices. They have been tested by people with Parkinson’s in a previous study and no problems have been reported.

**What are the possible benefits of taking part?**

The information gathered will contribute to the development of non-invasive mobile technology devices that can be used for monitoring of symptoms related to the management of Parkinson’s. We hope that in the future the PD_Manager system will help with the management of many people with Parkinson’s.

**Will our taking part in the study be kept confidential?**

Information collected through the PD_Manager devices will be stored in a public cloud platform provided by Biotronics3D. The Information Governance of Biotronics3D provides the highest levels of security and reliability to ensure the confidentiality of patient information.

All the information collected from you for the research will be analysed by the research team in England. It will be kept strictly confidential as required by the Data Protection Act (1998). All data collection forms will have a unique identification number, specific to you. No names or personal information will be kept on or stored with these forms. Information will be kept in locked filing cabinets and password protected computers, in a room with restricted access at the University of Surrey. Data are stored on secure University servers operated in accordance with the data protection act. The information collected will be shared with researchers in the other 5 European countries involved in the PD Manager project. Under no circumstances will any of your personal details be passed onto third parties, or appear in any reports on this study.

**What will happen if we do not want to carry on with the study?**

You can withdraw from the study at any time, without giving any reason, and this will in no way affect the usual care that the person with Parkinson’s receives. Provided you inform us in time that you do not want your data to be used, we will destroy it and it will be excluded from the analysis.

**What will happen to the results of the research study?**

The results of this study will be presented to the funder as a report. The research team will write papers for publications in journals and make presentations at conferences, to help influence the development of future services for people with Parkinson’s. Summaries of the study’s results will be available from the project’s website <http://www.parkinson-manager.eu/> or the research team.

**Who has reviewed the study?**

All research in the National Health Service is reviewed and approved by a Research Ethics Committee, which is responsible for protecting your interests and safety. This study has also received favourable ethical opinion from an ethics committee. .

**What if there is a problem?**

If you have a concern about any aspect of this study, you should speak to the researchers who will do their best to answer your questions. The University has in force the relevant insurance policies which apply to this study. In addition, the Sponsor has made arrangements, in the event of harm where no legal liability arises, for “non-negligent harm” claims. If you wish to complain, or have any concerns about any aspect of the way you have been treated during the course of this study then you should contact the people named below.

The Principal Investigator is Professor Heather Gage, Surrey Health Economics Centre, University of Surrey, Guildford, GU2 7XH. Email: [h.gage@surrey.ac.uk](mailto:h.gage@surrey.ac.uk) ; Telephone: 01483 68 6948

The Research Fellow is Morro Touray, Surrey Health Economics Centre, University of Surrey, Guildford, GU2 7XH. Email: [m.touray@surrey.ac.uk](mailto:m.touray@surrey.ac.uk) ; Telephone: 01483 68 8614.

Professor Joao Santos Silva, Head of School of Economics, University of Surrey Guildford GU2 7XH, England. Email: [jmcss@surrey.ac.uk](mailto:jmcss@surrey.ac.uk). Tel: 44 (0)1483 686956

**Thank you for taking the time to read this information.**

**If you have decided to take part in the pilot study, you contribution is much appreciated.**

**Please contact the research team by either:**

- completing the attached sheet and returning it in the stamped addressed envelope provided, or
- telephoning Morro Touray on **07842 777 688**
- sending an email to the principal investigator: [h.gage@surrey.ac.uk](mailto:h.gage@surrey.ac.uk) or the research fellow: [m.touray@surrey.ac.uk](mailto:m.touray@surrey.ac.uk)

APPENDIX C(i)


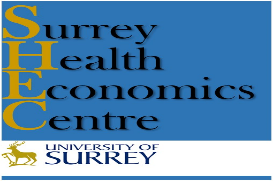


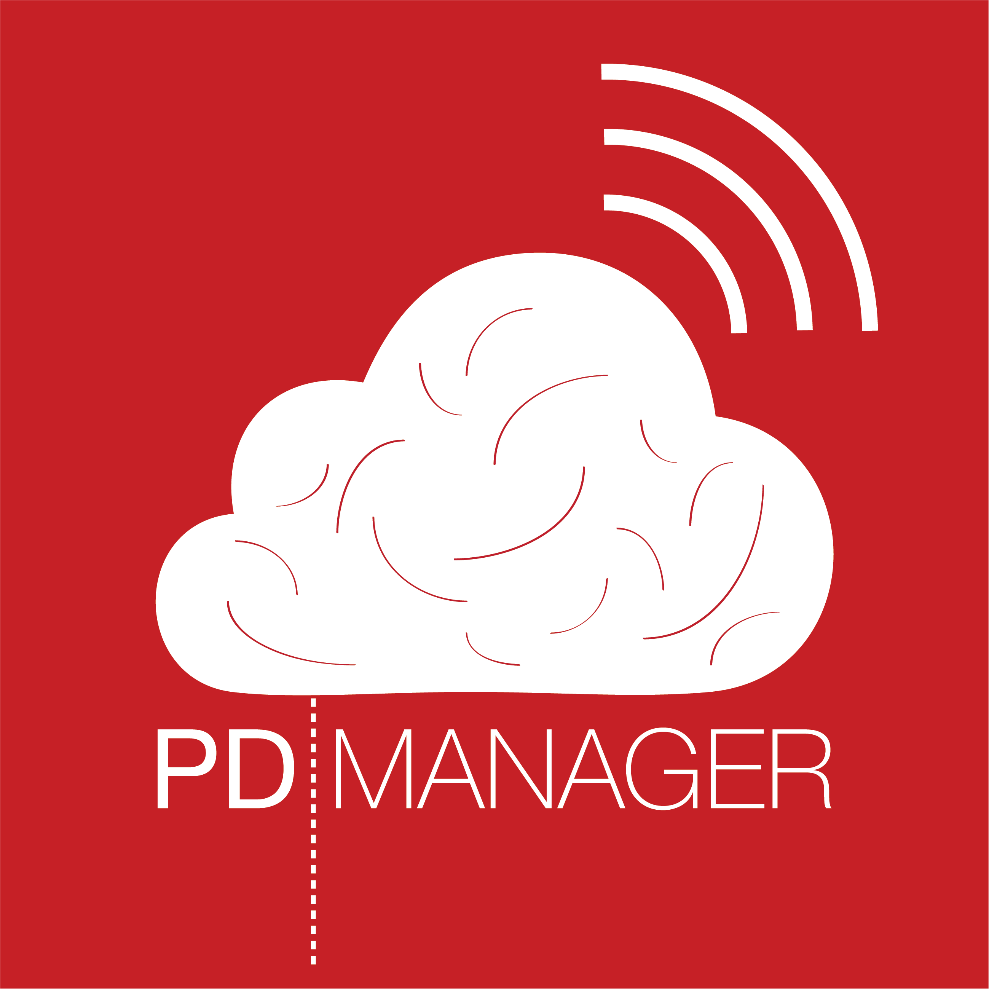


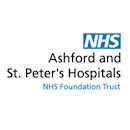


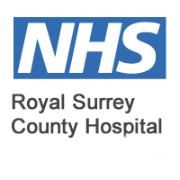


[
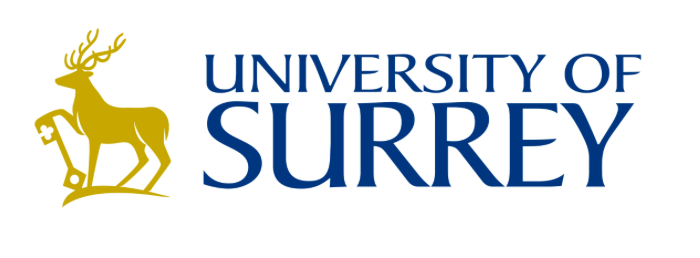
](http://www.google.co.uk/url?sa=i&rct=j&q=&esrc=s&source=images&cd=&cad=rja&uact=8&ved=0ahUKEwiosZXx2KjRAhVD0hoKHRF6CgoQjRwIBw&url=http://personal.ee.surrey.ac.uk/Personal/P.Barnaghi/&psig=AFQjCNHZRrZ1UmgFHoacYK82NIvMSmcI3A&ust=1483626317862176)

Form Number: 02

**PD_Manager: Consent Form for Person with Parkinson’s**

|  | | Please initial boxes |
| --- | --- | --- |
| 1 | I voluntarily agree to take part in the study. |  |
| 2 | I confirm that I have read and understood the information sheet dated _________ (version ___). I have had the opportunity to ask questions on all aspects of the study, and have had these answered satisfactorily. |  |
| 3 | I understand that I will be randomly assigned to receive either PD Manager devices or a diary as described to me to gather relevant data to support treatment decisions. I understand that the trial is only for a limited period of time. |  |
| 4 | I understand that I will be asked to visit the hospital for assessment by the research nurse or clinician on two occasions and any related reasonable travel expenses will be reimbursed. |  |
| 5 | I understand that my participation is voluntary and that I am free to withdraw at any time without giving any reason, and without my medical care or legal rights being affected. |  |
| 6 | I agree that if I withdraw from the study for any reason, all of my data collected up to that point may be retained and used unless I specifically request on time that it is disregarded. |  |
| 7 | I agree to my General Practitioner (GP) being informed about my participation in the study. |  |
| 8 | I consent to my personal data being used for the study as detailed in the information sheet. I understand that all personal data relating to volunteers is held and processed in the strictest confidence, and in accordance with the Data Protection Act (1998). |  |
| 9 | I confirm that I have read and understood the above and freely consent to participating in this study. I have been given adequate time to consider my participation and agree to comply with the instructions of the study. |  |

____________________________ _____________________________ ____________________________

Name of Participant Signature Date and Time

____________________________ _____________________________ ____________________________

Name of Person taking consent Signature Date and Time

___________________________ ____________________________ _____________________________

Name of Witness) Signature Date and Time

*(Copies: 1 for participant, 1 for GP to be kept in medical note,) and original for Researcher)*

APPENDIX C(ii)


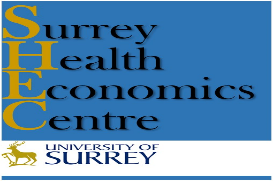


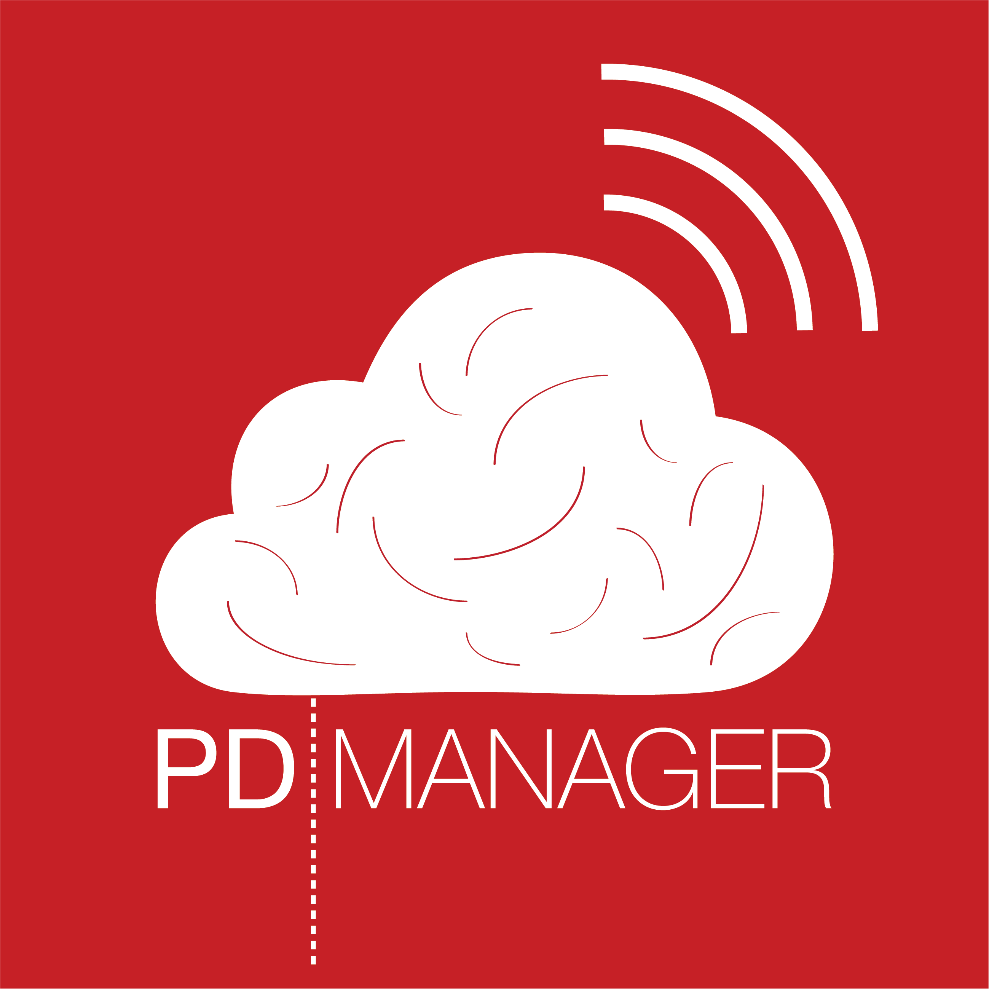


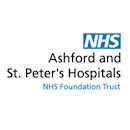


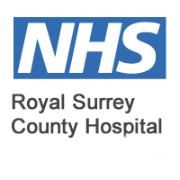


[
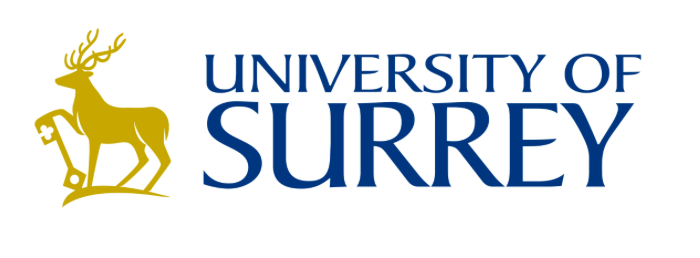
](http://www.google.co.uk/url?sa=i&rct=j&q=&esrc=s&source=images&cd=&cad=rja&uact=8&ved=0ahUKEwiosZXx2KjRAhVD0hoKHRF6CgoQjRwIBw&url=http://personal.ee.surrey.ac.uk/Personal/P.Barnaghi/&psig=AFQjCNHZRrZ1UmgFHoacYK82NIvMSmcI3A&ust=1483626317862176)

Form Number: 03

**PD_Manager: Consent Form for family member or friend of Person with Parkinson’s**

|  | | Please initial boxes |
| --- | --- | --- |
| 1 | I voluntarily agree to take part in the study and to assist the person with Parkinson’s with the PD_Manager devices or symptom diary. |  |
| 2 | I confirm that I have read and understood the information sheet dated _________ (version ___). I have had the opportunity to ask questions on all aspects of the study, and have had these answered satisfactorily. |  |
| 3 | I understand that the person with Parkinson’s that I live with will be randomly assigned to receive either PD Manager devices or a diary as described to gather relevant data to support treatment decisions. I understand that the trial is only for a limited period of time. |  |
| 4 | I understand that I will be asked to visit the hospital for assessment by the research nurse or clinician on two occasions. |  |
| 5 | I understand that my participation is voluntary and that I am free to withdraw at any time without giving any reason, and without my medical care or legal rights being affected. |  |
| 6 | I agree that if I withdraw from the study for any reason, all of my data collected up to that point may be retained and used unless I specifically request that it is disregarded. |  |
| 7 | I consent to my personal data being used for the study as detailed in the information sheet. I understand that all personal data relating to volunteers is held and processed in the strictest confidence, and in accordance with the Data Protection Act (1998). |  |
| 8 | I confirm that I have read and understood the above and freely consent to participating in this study. I have been given adequate time to consider my participation and agree to comply with the instructions of the study. |  |

____________________________ _____________________________ ____________________________

Name of Participant Signature Date and Time

____________________________ _____________________________ ____________________________

Name of Person taking consent Signature Date and Time

___________________________ ____________________________ _____________________________

Name of Witness Signature Date and Time

*(Copies: 1 for participant, 1 for GP to be kept in medical note,) and original for Researcher)*

Appendix E

**PD_Manager. Pilot testing of devices to assist with symptom management for people with Parkinson’s.**

**Information sheet for health professionals**

We are inviting health professionals that care for people with Parkinson’s to take part in a research study. Before you decide, please take the time to carefully read the information below. This explains why the research is being done and what taking part would involve for you.

If you decide you would like to volunteer to take part, please respond to the research team using the contact details that are given at the end of this information sheet.

**What is the PD_Manager project?**

People with moderate to advanced Parkinson’s often have fluctuating symptoms which can make it difficult for health professionals to customise treatment to their needs.

New technologies have been designed in the PD_Manager project to track Parkinson’s symptoms in real time and provide a continuous record for health professionals. The research proposed aims to find out if this information can help doctors and Parkinson’s nurses to manage peoples’ symptoms better. The technologies include wristbands and insoles. The devices are linked to a smartphone which will be loaded with some ‘apps’ designed to help people with Parkinson’s manage their condition. The smartphone will also be used to store information about symptom fluctuations and to send it to their Parkinson’s doctor or nurse.

PD_Manager is funded by the European Commission through the Horizon 2020 Research and Innovation programme. Researchers from six European countries are involved.

**What is the purpose of this study?**

The purpose of this pilot study is to test the PD_Manager system with a small number of people with Parkinson’s. We want to see if people with Parkinson’s and the family member or friend they live with find the devices acceptable. We will also ask doctors if they find the information that is generated through PD_Manager helpful when they are deciding on treatments. To help us assess how useful the PD_Manager system is, we are comparing it with a symptom diary which we will ask participants to complete on a daily basis.

**Why have I been invited?**

You have been invited to participate in the pilot study because you are a doctor or nurse specialising in the care of people with Parkinson’s. Participation is completely voluntary.

**What will participation in the study involve for me?**

If you are a Parkinson’s nurse and you volunteer to participate in the pilot study, you will be asked to help with gathering background information from 20 people with Parkinson’s, and their live-in carers (family members or friends). The participants will be asked to meet you at your hospital clinic. When the baseline information is gathered, the researcher will randomly assigned the participant to receive either the PD_Manager devices or the symptom diary. The devices or diary will then be provided to the participant (with full information and a telephone helpline to the research team). Participants will be asked to test the devices or diary for two weeks. Prior to leaving the clinic, you will help to make an appointment for the participants to return at the end of the two weeks to meet with their Parkinson’s doctor.

If you are a Parkinson’s doctor and you volunteer to take part in the trial, you will be asked to complete a baseline questionnaire to provide information on your qualifications, current role and attitudes to technology. Your role in the research will be to hold a consultation with each person with Parkinson’s (and live-in carer) at the end of the two week trial period to review the information that has been collected through the devices or the diary (depending on group allocation). If you judge that changes in treatment, or referrals to other health professionals, are needed, we will ask you to report these to us.

We expect each doctor to review a maximum of ten participant dyads, some of whom will have tested the PD_Manager devices and some who will have used the symptom diary. A researcher will be on hand to ask participants for their feedback. The researcher will also ask you for your views on the strengths and limitations of the information generated by the devices and the diary, and for some background information about your past and present practice. If you initiate treatment or referrals, you may wish to inform the participant’s GP. We will seek permission from participants to inform their GPs that they are taking part in this study, and the group they are allocated to.

**What are the possible benefits of taking part?**

The information gathered will contribute to the development of non-invasive mobile technology devices that can be used for monitoring of symptoms related to the management of Parkinson’s. We hope that in the future the PD_Manager system will help with the management of many people with Parkinson’s.

**Will my taking part in the study be kept confidential?**

All the information collected for the research will be kept strictly confidential as required by the Data Protection Act (1998). All questionnaires will have a unique identification number, specific to the respondent. No names or personal information will be kept on or stored with these questionnaires. Information will be kept in locked filing cabinets and password protected computers, in a room with restricted access at the University of Surrey. Data will be stored on secure University servers. The information collected will be analysed to meet the aims of the study and will be shared with researchers in the other 5 European countries involved in the PD Manager project. Under no circumstances will any personal details of participants be passed onto third parties, or appear in any reports on this study.

Information collected through the PD_Manager devices will be stored in a public cloud platform provided by Biotronics3D. The servers where the information in the cloud platform is stored is based in the UK and operated in accordance with the data protection act. The Information Governance of Biotronics3D provides the highest levels of security and reliability to ensure the confidentiality of patient information.

**What will happen if I do not want to carry on with the study?**

You can withdraw from the study at any time, without giving any reason. Provided you inform us in time that you do not want your information to be used, we will exclude it from the analysis.

**What will happen to the results of the research study?**

The results of this study will be presented to the funder as a report. The research team will write papers for publications in journals and make presentations at conferences, to help influence the development of future services for people with Parkinson’s. Summaries of the study’s results will be available from the project’s website <http://www.parkinson-manager.eu/> or the research team.

**Who has reviewed the study?**

All research in the National Health Service is reviewed and approved by a Research Ethics Committee, which is responsible for protecting your interests and safety.

**What if there is a problem?**

If you have a concern about any aspect of this study, the researchers will do their best to answer your questions. The University has in force the relevant insurance policies which apply to this study. In addition, the Sponsor has made arrangements, in the event of harm where no legal liability arises, for “non-negligent harm” claims. If you wish to complain, or have any concerns about any aspect of the way you have been treated during the course of this study then you should follow the instructions given above.

The Principal Investigator is Professor Heather Gage, Surrey Health Economics Centre, University of Surrey, Guildford, GU2 7XH. Email: [h.gage@surrey.ac.uk](mailto:h.gage@surrey.ac.uk) ; Telephone: 01483 68 6948

The Research Fellow is Morro Touray, Surrey Health Economics Centre, University of Surrey, Guildford, GU2 7XH. Email: [m.touray@surrey.ac.uk](mailto:m.touray@surrey.ac.uk) ; Telephone: 01483 68 8614.

Professor Joao Santos Silva, Head of School of Economics, University of Surrey Guildford GU2 7XH, England. Email: [jmcss@surrey.ac.uk](mailto:jmcss@surrey.ac.uk). Tel: 44 (0)1483 686956

**Thank you for taking the time to read this information.**

**If you have decided to take part in the pilot study, you contribution is much appreciated.**

**Please contact the research team by either:**

- telephoning Morro Touray on **07842 777 688**
- sending an email to the principal investigator: [h.gage@surrey.ac.uk](mailto:h.gage@surrey.ac.uk) or the research fellow: [m.touray@surrey.ac.uk](mailto:m.touray@surrey.ac.uk)

APPENDIX F


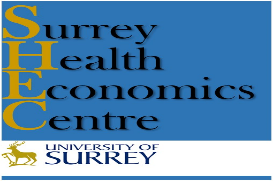


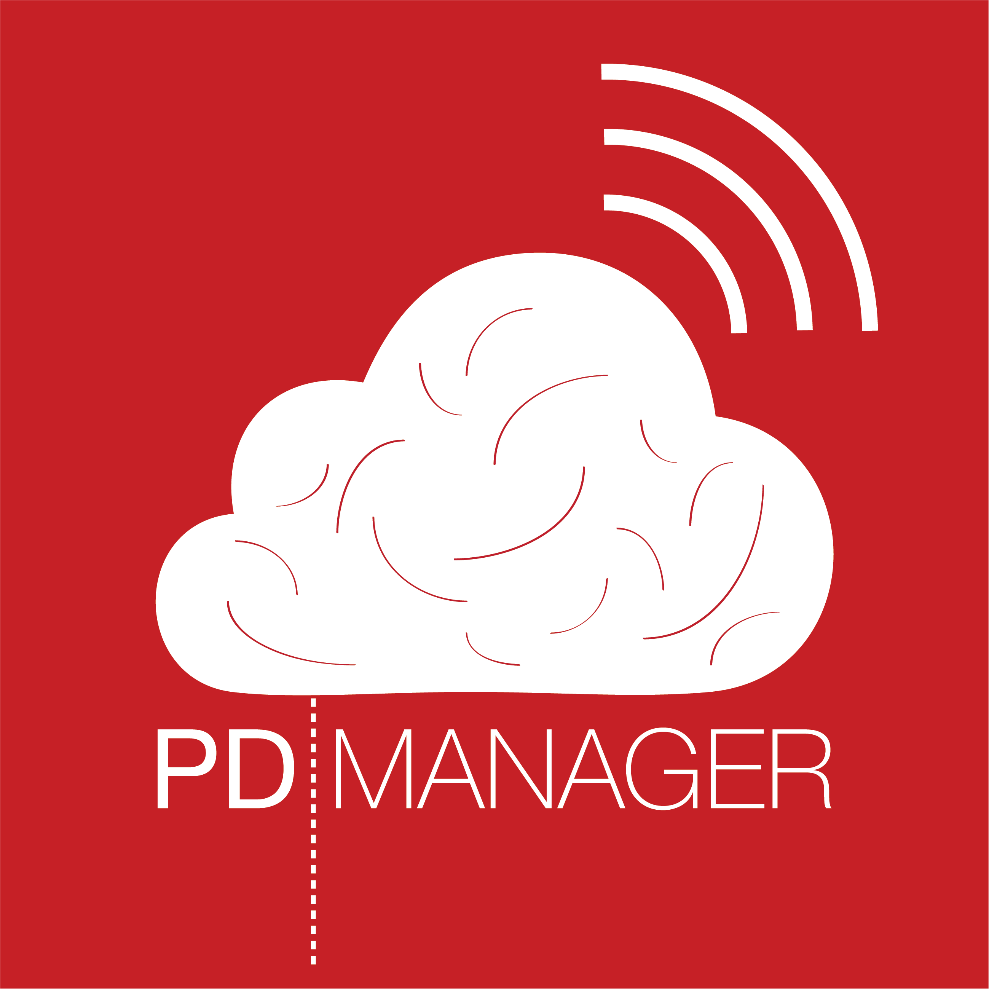


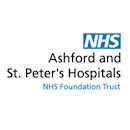


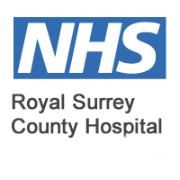


[
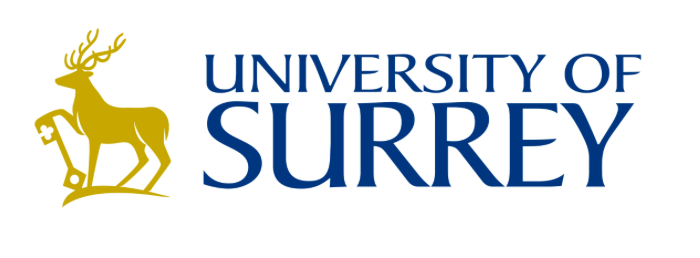
](http://www.google.co.uk/url?sa=i&rct=j&q=&esrc=s&source=images&cd=&cad=rja&uact=8&ved=0ahUKEwiosZXx2KjRAhVD0hoKHRF6CgoQjRwIBw&url=http://personal.ee.surrey.ac.uk/Personal/P.Barnaghi/&psig=AFQjCNHZRrZ1UmgFHoacYK82NIvMSmcI3A&ust=1483626317862176)

Form Number: 06

**PD_Manager: Consent Form for Health Professionals**

**BASELINE: Parkinson’s nurse: ………………………………….. Date: …………………………………**

|  | | Please initial boxes |
| --- | --- | --- |
| 1 | I voluntarily agree to take part in the study. |  |
| 2 | I confirm that I have read and understood the information sheet dated _________ (version ___). I have had the opportunity to ask questions on all aspects of the study, and have had these answered satisfactorily. |  |
| 3 | I understand that as Parkinson’s nurse / doctor, I will be asked to review people with Parkinson’s and their live-in carers at the start / end of the trial and provide feedback to the research team as requested. |  |
| 4 | I understand that my participation is voluntary and that I am free to withdraw at any time without giving any reason. |  |
| 5 | I agree that if I withdraw from the study for any reason, all of my data collected up to that point may be retained and used unless I specifically request on time that it is disregarded. |  |
| 6 | I consent to my personal data being used for the study as detailed in the information sheet. I understand that all personal data relating to volunteers is held and processed in the strictest confidence, and in accordance with the Data Protection Act (1998). |  |
| 7 | I confirm that I have read and understood the above and freely consent to participating in this study. I have been given adequate time to consider my participation and agree to comply with the instructions of the study. |  |

____________________________ _____________________________ ____________________________

Name of Participant Signature Date and Time

____________________________ _____________________________ ____________________________

Name of Person taking consent Signature Date and Time

___________________________ ____________________________ _____________________________

Name of Witness (*optional*) Signature Date and Time

*(Copies: 1 for participant, 1 [original] for researcher site file)*
